# Genetic and ultrasonographic analyses of fetuses with 1q21.1q21.2 microdeletion/microduplication: a retrospective study

**DOI:** 10.1186/s12920-023-01618-4

**Published:** 2023-08-23

**Authors:** Nan Guo, Huili Xue, Bin Liang, Hailong Huang, Meiying Cai, Liangpu Xu

**Affiliations:** Medical Genetic Diagnosis and Therapy Center, Fujian Maternity and Child Health Hospital College of Clinical Medicine for Obstetrics & Gynecology and Pediatrics, Fujian Medical University, Fujian Key Laboratory for Prenatal Diagnosis and Birth Defect, Fuzhou, China

**Keywords:** 1q21.1q21.2 microdeletion/microduplication, Genetic analysis, Ultrasonographic analysis, Chromosomal microarray analysis, Penetrant genetic mutation

## Abstract

**Background:**

1q21.1q21.2 microdeletions/microduplications are rare and incompletely penetrant genetic mutations, and only a few reports regarding their prenatal diagnosis are currently available. Here, we analyzed the ultrasonographic phenotypic characteristics of fetuses with these mutations to improve the understanding, diagnosis, and screening of these mutations during gestation.

**Methods:**

We retrospectively analyzed 8700 cases of pregnant women who underwent invasive prenatal screening by karyotyping and chromosomal microarray analysis (CMA) between November 2016 and November 2021.

**Results:**

CMA revealed copy number changes in the 1q21.1q21.2 region of eleven fetuses, of which five had microdeletions and six had microduplications. These eleven fetuses exhibited variable ultrasonographic phenotypes. Of the five fetuses with the microdeletion, one exhibited a right-dominant heart, permanent right umbilical vein, and mild tricuspid regurgitation, another showed thickened nuchal translucency, and the remaining three had normal ultrasound phenotypes. Two of the six cases with 1q21.1q21.2 microduplication had structural malformations; one of them had a bilateral subependymal cyst, neck mass, and enlarged cardiothoracic ratio, while the other had right ventricular hypoplasia. Of the remaining four cases, two exhibited nasal bone dysplasia, one showed measurement slower than that during menopause and mild tricuspid regurgitation, and another did not show any notable abnormality in ultrasound examination. Among the eleven cases of 1q21.1q21.2 microdeletion/microduplication, only the parents of two fetuses underwent pedigree verification. The parents of these two fetuses with 1q21.1q21.2 microdeletion syndrome chose to continue the pregnancy, and all aspects of postnatal follow-up were normal. The parents of the other nine fetuses refused pedigree verification; of these cases, four cases terminated, and five cases continued the pregnancies. The five continued pregnancies were followed up after birth; no abnormalities were found.

**Conclusions:**

Fetuses with 1q21.1q21.2 microdeletion/microduplication show different ultrasound characteristics and may have congenital heart disease, thickened nuchal translucency, and nasal bone dysplasia or show no notable abnormalities in an ultrasound examination. Our study highlights that CMA as a powerful diagnostic tool for these diseases can provide an accurate genetic diagnosis, while improving prenatal diagnosis standards.

## Background

Genomic disorders are caused by alterations in the human genome. The q21.1q21.2 region of chromosome 1 is rich in low-copy repeats (LCRs), which increase the probability of non-allelic homologous recombination (NAHR) in this region [1]. As NAHR can cause chromosomal microdeletions and microduplications, the 1q21.1q21.2 region is especially prone to such events. The clinical manifestations in patients with complete 1q21.1q21.2 microdeletion syndrome (MIM:612,474) penetrance are diverse. Patients may exhibit mild clinical manifestations, or microcephaly, special facial features, mental retardation, developmental delay, congenital heart disease, and other clinical manifestations [2–5]. Fragment duplication in the 1q21.1q21.2 region results in microduplication syndrome (MIM:612,475). Patients with incomplete 1q21.1q21.2 microduplication penetrance have similar clinical manifestations and may exhibit congenital heart disease, macrocephaly, special facial features, abnormal behavior, autism, mental retardation, and other clinical manifestations [6–9].

With the advancement of prenatal screening and molecular detection technology and the widespread use of chromosomal microarray analysis (CMA) in prenatal diagnosis, an increasing number of microdeletion/microduplication syndromes have been discovered [10]. The q21.1q21.2 region of chromosome 1 contains multiple clusters of fragment repeats or LCRs, making it susceptible to repeated rearrangements. Some researchers believe that the 1q21.1 chromosomal region can be divided into two distinct regions, namely the proximal region, which extends from BP2 to BP3, and the distal region, which extends from BP3 to BP4. The distal region includes key genes such as *GJA5* and *GJA8. GJA5* encodes cardiac connexin 40, which plays a key role in cell adhesion and intercellular communication [11, 12], and defects in *GJA5* have been associated with congenital heart disease [7, 13–15]. The *GJA8* gene encodes connexin 50, and defects in this gene have been associated with autosomal dominant type 1 congenital cataract [16]. The proximal regions of 1q21.1 are a susceptibility factor for thrombocytopenia-absent radius (TAR) syndrome, which is associated with the *RBM8A* gene. This gene encodes the Y14 protein, which is one of the four components of the exon-junction complex, and is involved in basic cellular functions, such as nuclear export and subcellular localization of specific transcripts, translational enhancement, and nonsense-mediated RNA decay. In addition, other candidate genes related to TAR include *PIAS3*, a regulator of hematopoietic growth factor signaling, and *LIX1L*, which is involved in limb development-related regulation. Early reports on individuals with TAR syndrome noted a 7% significant developmental delay incidence; however, it is unclear whether these proximal microdeletions have other phenotypic consequences.

The 1q21.1q21.2 microdeletion or microduplication event is a rare chromosomal abnormality, with only a few reported cases diagnosed during prenatal development [17]. Prenatal genetic counselling is challenging as these mutations may cause phenotypic diversity or no notable symptoms in the prenatal stage, although clinical manifestations may appear post-birth. Here, we present five cases of 1q21.1q21.2 microdeletion and six cases of 1q21.1q21.2 microduplication. We analyzed the prenatal clinical manifestations of these cases to improve our understanding of microduplication/microdeletion syndromes.

## Methods

### Patients

Between November 2016 and November 2021, 20,000 pregnant women underwent prenatal genetic screening at the Fujian Provincial Maternal and Child Health Care Hospital, of which, 8700 women underwent karyotyping and CMA. The average age and gestational age of the pregnant women were 28 ± 5 years and 23 ± 1 weeks, respectively.

### Karyotype analysis

Amniocentesis (16–24 weeks) and umbilical cord blood puncture (> 24 weeks) were performed using ultrasonography. When cell growth in the collected amniotic fluid sample was vigorous, colchicine was added to inhibit mitosis. The cells were then dissociated with trypsin and harvested. Next, the cells were treated with a hypotonic solution, fixed, and subjected to G-banding karyotype analysis. In the case of the umbilical cord blood samples, colchicine was added to the samples after three days of culture. Thirty cells were counted in each case; the count was increased to 100 if chimeras were found.

### CMA

Fetal genomic DNA was extracted from cord blood (1 mL) and amniotic fluid (10 mL) using DNA extraction kits from Qiagen (USA) and BioChain (USA), respectively. The DNA of fetuses was then subjected to CMA. The genomic DNA samples were purified, hybridized with microarrays, and analyzed in accordance with the standard procedures provided by Affymetrix. A genome-wide CytoScan™ HD chip (Affymetrix, USA) with a single-nucleotide polymorphism (SNP) probe was used. The corresponding ChAS software and related bioinformatics methods were used to analyze the CMA results. Copy number variations (CNVs) were determined using the scatter plot distribution of the DNA fragment copy number. We compared the CNVs using reference databases, including the Database of Genomic Variants (DGV) (http://projects.tcag.ca/variation), DECIPHER (http://www.sanger.ac.uk/PostGenomics/decipher), Online Mendelian Inheritance in Man (OMIM) (http://www.omim.org), and University of California Santa Cruz (UCSC) databases (http://www.genome.UCSC.edu/). CNVs were classified as pathogenic, likely pathogenic, benign, likely benign, and those with a variant of uncertain significance [18].

### Obstetric follow-up

Follow-up with the parents of all fetuses was carried out via phone call to determine pregnancy outcomes, diagnoses, and postnatal care.

## Results

### Karyotype analysis and CMA

Among the 8700 fetuses who simultaneously underwent karyotype analysis and CMA, eleven fetuses had abnormal CNVs in the 1q21.1q21.2 region, but the karyotype analysis yielded normal results (the abnormality rate was approximately 0.1%). Of these eleven fetuses, six had copy number microduplication and five had copy number microdeletion in the 1q21.1q21.2 region.

### Molecular characteristics and ultrasonic phenotypes of fetuses with 1q21.1q21.2 microdeletion

**Fig. 1:**
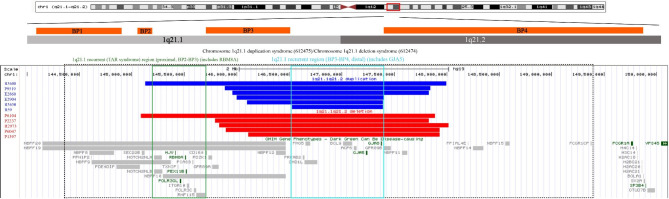
Chromosomal microarray analysis results revealed 1q21.1q21.2 microdeletion/microduplication in eleven fetuses. Five fetuses had 1q21.1q21.2 microdeletion (with the fragment size ranging between1.8–2.8 Mb) and six fetuses had microduplication (with the fragment size ranging between 0.86–2.9 Mb). These alterations occurred in the 1q21.1q21.2 region, which included the *GJA5* and *GJA8* genes. The dotted box indicates the genomic location of the 1q21.1 chromosome microdeletions/microduplications in the Online Mendelian Inheritance in Man (OMIM) database, green box indicates the 1q21.1 recurrent region (BP2-BP3) in the ClinGen database, and blue box represents the 1q21.1 recurrent region (BP3-BP4) in the ClinGen database

The size of the microdeletion fragments in the 1q21.1q21.2 region in the five fetuses ranged between 1.8 and 2.8 Mb. In four cases, the microdeletions included the *GJA5* and *GJA8* genes, which are within the 1q21.1 recurrent region (BP3-BP4) (Fig. 1). The fifth case additionally harbored a microdeletion in the 1q21.1 recurrent region (BP2-BP4). In ultrasound examination, the fetus that harbored the BP2-BP4 region microdeletion exhibited a right-dominant heart, permanent right umbilical vein, and mild tricuspid regurgitation, while another fetus showed thickened nuchal translucency. The remaining three fetuses showed normal ultrasound phenotypes. After genetic counseling, the parents of only two out of the five fetuses with microdeletions agreed to undergo pedigree verification, which confirmed paternal inheritance (Table 1).

**Table 1:** Molecular characteristics and indication for the invasive diagnosis of a fetus with 1q21.1q21.2 microdeletion

Case	Indication for invasive diagnostic	CMA	Size (Mb)	Breakpoint regions	Inheritance	Pregnancy outcome
P1397	Balanced translocation of paternal chromosomes(45,XY,der(14;21)(q10; q10))	arr[GRCh37]1q21.1q21.2(146,106,723_147,933,973)x1	1.8	1q21.1recurrentregion (BP3-BP4)	Paternal	Healthy
R2973	Fetal ultrasound abnormality: thickened nuchal translucency	arr[GRCh37]1q21.1q21.2(145,829,473_148,016,122)x1	2.2	1q21.1 recurrent region (BP3-BP4)	Refused	Healthy
P6104	Fetal ultrasound abnormality: right-dominant heart, permanent right umbilical vein, and mild tricuspid regurgitation	arr[GRCh37]1q21.1q21.2(145,084,525_147,885,600)x1	2.8	1q21.1 recurrent region (BP2-BP4)	Paternal	Normal appearance (No ultrasound examination)
P2237	High risk of Down syndrome screening	arr[GRCh37]1q21.1q21.2(145,792,037_147,830,830)x1	2.0	1q21.1 recurrent region (BP3-BP4)	Refused	Healthy
P6047	Advanced age	arr[GRCh37]1q21.1q21.2(145,895,746_147,933,973)x1	2.0	1q21.1 recurrent region (BP3-BP4)	Refused	TP

CMA, chromosomal microarray analysis;TP, termination of pregnancy

### Molecular characteristics and ultrasonic phenotypes of fetuses with the 1q21.1q21.2 microduplication

The size of the microduplication fragments in the 1q21.1q21.2 region in the six fetuses ranged between 0.86 and 2.9 Mb. In five cases, the microduplications included the *GJA5* and *GJA8* genes, which are within the 1q21.1 recurrent region (BP3-BP4) (Fig. 1). The sixth case additionally harbored a microduplication in the 1q21.1 recurrent region (BP2-BP4). Two of the six cases showed structural malformations–one case had a bilateral subependymal cyst, neck mass, and enlarged cardiothoracic ratio, while the other had right ventricular hypoplasia (Table 2). The most common symptom in two of these four fetuses was nasal dysplasia. Of the two remaining fetuses that exhibited atypical ultrasonography, one showed mild tricuspid regurgitation and the other (the fetus that harbored the BP2-BP4 microduplication), showed no notable abnormality with ultrasound examination. The fetus with a normal ultrasound phenotype was diagnosed via prenatal diagnosis because of the mother’s advanced age. After genetic counseling, the parents of all six fetuses harboring 1q21.1q21.2 microduplications refused pedigree verification.

**Table 2:** Molecular characteristics and indication for the invasive diagnosis of a fetus with 1q21.1q21.2 microduplication

Case	Indication for invasive diagnostic	CMA	Size (Mb)	Breakpoint regions	Inheritance	Pregnancy outcome
R3608	Advanced age	arr[GRCh37]1q21.1q21.2(145,124,436_147,995,251)x3	2.9	1q21.1 recurrent region (BP2-BP4)	Refused	Healthy
R3650	Fetal ultrasound abnormality: nasal dysplasia	arr[GRCh37]1q21.1q21.2(146,096,700_147,391,923)x3	1.3	1q21.1 recurrent region (BP3-BP4)	Refused	Died 2 days after birth
P9519	Fetal ultrasound abnormality: each measurement value less than the duration of menopause, and mild tricuspid regurgitation	arr[GRCh37]1q21.1q21.2(145,886,339_147,844,777)x3	1.9	1q21.1 recurrent region (BP3-BP4)	Refused	TP
E2860	Fetal ultrasound abnormality: bilateral subependymal cysts, neck mass, and enlarged cardiothoracic ratio	arr[GRCh37]1q21.1q21.2(145,958,361_147,830,830)x3	1.8	1q21.1 recurrent region (BP3-BP4)	Refused	TP
E2904	Fetal ultrasound abnormality: right ventricular hypoplasia	arr[GRCh37]1q21.1q21.2(145,995,176_147,398,268)x3	1.4	1q21.1 recurrent region (BP3-BP4)	Refused	TP
R59	Fetal ultrasound abnormality: nasal dysplasia	arr[GRCh37]1q21.1q21.2(146,525,270_147,391,923)x3	0.86	1q21.1 recurrent region (BP3-BP4)	Refused	Healthy

CMA, chromosomal microarray analysis; TP, termination of pregnancy

### Obstetric outcomes

In the eleven 1q21.1q21.2 microdeletion or microduplication cases, the parents of four fetuses chose to terminatethe pregnancy, while the parents of the remaining seven fetuses chose to continue after adequate genetic counseling, and follow-up was performed after normal deliveries. Except for one patient who died two days after birth, abnormalities were not found in the other patients from eight months to four years of the children’s age, based on telephonic follow-ups.

## Discussion

The clinical manifestations of 1q21.1q21.2 microdeletion/microduplication are diverse, and its penetrance was incomplete. Patients may show an absence of clinical manifestations, mild manifestations [19, 20], craniofacial abnormalities, mental retardation, developmental delay, congenital heart disease, abnormal behavior, autism, and epilepsy [19, 21–25]. The aforementioned characteristics represent clinical phenotypes of 1q21.1q21.2 microdeletions/microduplications in children and adults. Currently, only a few studies report the prenatal diagnosis of these conditions. Chen et al. [26] reported that a fetus with a 1q21.1q21.2 microdeletion had oligohydramnios, bilateral renal dysplasia, and polydactyly of the left foot. Bouariuet al. [27] reported a rare case of an allantoic cyst with a patent urachus in a fetus with a 1q21.1q21.2 microdeletion. Here, we reported five cases of 1q21.1q21.2 microdeletion. Four of the cases harbored microdeletions in the *GJA5* and *GJA8* genes, which are included in the 1q21.1 recurrent region (BP3-BP4). One case additionally harbored a microdeletion in the TAR region within the 1q21.1 recurrent region (BP2-BP4). This case (with the additional TAR region microdeletion) showed thickened nuchal translucency, and another showed a right-dominant heart, permanent right umbilical vein, and mild tricuspid regurgitation, which were supplemented by the prenatal ultrasound phenotypes of fetuses harboring 1q21.1q21.2 microdeletions. Zhang et al. [6] reported abnormal nasal bone and ventricular septal defects in a fetus with 1q21.1q21.2 microduplication. Fu et al. [28] reported 1q21.1q21.2 microduplication in a fetus with a ventricular septal defect, pulmonary atresia, and a persistent left superior vena cava. In this study, we identified six fetuses with 1q21.1q21.2 microduplication. Five cases harbored microduplications in the *GJA5* and *GJA8* genes, which were within the 1q21.1 recurrent region (BP3-BP4). One case additionally harbored a microduplication in the TAR region within the 1q21.1 recurrent region (BP2-BP4). This case (with the additional TAR region microduplication) had a normal ultrasound phenotype. The ultrasound phenotype of two fetuses with 1q21.1 microduplication in the recurrent region (BP3-BP4) was nasal dysplasia, which was consistent with previous findings. Furthermore, the ultrasound phenotypes of the fetuses with 1q21.1q21.2 microduplication in region BP3-BP4 included bilateral subependymal cysts, neck mass, enlarged cardiothoracic ratio, and right heart dysplasia. The abnormal expression of *GJA5* and its flanking gene *GJA8* has previously been associated with congenital heart disease [29]; hence, the cardiac abnormalities identified in our study may be associated with these two genes. The three cases described above corroborate the prenatal clinical manifestations of 1q21.1q21.2 microduplication in a fetus. Some fetuses with 1q21.1q21.2 microdeletion/microduplication lack significant clinical manifestations before birth, possibly due to insufficient penetrance. However, even though the prenatal ultrasound examination may not reveal any abnormality, a fetus with 1q21.1q21.2 microdeletion/microduplication may exhibitlow intelligence, autism, and abnormal behavior after birth, which pose significant challenges for prenatal genetic counseling.

In this study, all but two sets of parents (of two fetuses with 1q21.1q21.2 microdeletion) refused to undergo pedigree verification. Paternal heredity could be determined in the cases where parents agreed to pedigree verification, and after receiving genetic counseling, these parents continued the pregnancies, and the postnatal follow-up revealed normal results in all aspects. The parents of four of the nine cases who refused pedigree verification terminated their pregnancies after genetic counseling, whereas the remaining five continued. Abnormalities were not found in these five fetuses with 1q21.1q21.2 microdeletion/microduplication in the follow-up after birth. Due to the incomplete penetrance and lack of specific clinical manifestations associated with the 1q21.1q21.2 microdeletion/microduplication region [29, 30], genetic counseling for its prenatal CMA detection remains a challenge for obstetricians and genetic counselors. In addition to imaging, other prenatal diagnostic techniques should be used to comprehensively evaluate the developmentof such fetuses, and various indicators of fetal growth and development should be evaluated regularly.

This study has a few limitations. First, we only identified eleven fetuses with 1q21.1q21.2 microdeletion/microduplication, which is a small sample size. Second, whole-exome sequencing was not performed on the eleven fetuses, thereby preventing additional pathogenic gene identification. Third, only two of the eleven cases could be verified by pedigree analysis. Thus, future studies with larger sample size, whole-exome sequencing analysis, and pedigree verification for more patients, if possible, are required to improve our understanding of patients with 1q21.1q21.2 microdeletion/microduplication.

## Conclusions

Fetuses with 1q21.1q21.2 microdeletions/microduplications can exhibit different ultrasound characteristics; they could have congenital heart disease, thickened nuchal translucency, and nasal bone dysplasia or they may not show any notable ultrasound abnormalities. The assessment of different clinical phenotypes can provide a valuable theoretical basis for elucidating the pathogenesis, diagnosis, and treatment of these diseases. CMA is a powerful diagnostic tool for these diseases and can provide an accurate genetic diagnosis, while improving the standard of prenatal diagnosis. Our findings indicate that prenatal diagnosis methods, including CMA, can rule out fetal chromosomal abnormalities in pregnant women with abnormal prenatal ultrasonography findings, an advanced age, and high screening risk.

## Data Availability

All data generated during and/or analyzed during the current study are available from the corresponding author on reasonable request.

## List of abbreviations

CMA: Chromosomal microarray analysis
CNV: Copy number variation
DGV: Database of Genomic Variants
LCRs: Low-copy repeats
NAHR: Non-allelic homologous recombination
OMIM: Online Mendelian Inheritance in Man
TAR: Thrombocytopenia-absent radius
UCSC: University of California Santa Cruz
